# Severe thrombocytopenia in a patient with hepatitis C treated with eltrombopag from off-label drug use to on-label drug use: a case report

**DOI:** 10.1186/1752-1947-8-303

**Published:** 2014-09-10

**Authors:** Hassan A Al-Jafar, Jameela Al-Khaldi, Ahmad Alduaij, Khalifa Al-Banwan

**Affiliations:** 1 Hematology Department, Amiri Hospital, Gulf Street, Kuwait City, Kuwait; 2 Gastroenterology Department, Amiri Hospital, Gulf Street, Kuwait City, Kuwait; 3 Histopathology Department, Amiri Hospital, Gulf Street, Kuwait City, Kuwait; 4 Microbiology Department, Amiri Hospital, Gulf Street, Kuwait City, Kuwait

**Keywords:** Hepatitis C virus, Off-label drug use, Peginterferon, Thrombocytopenia, Eltrombopag

## Abstract

**Introduction:**

Off-label drug use refers to drug use beyond the specifications authorized for marketing. Eltrombopag is a new thrombopoietin receptor agonist which was used successfully in this critical case of thrombocytopenia associated with hepatitis C infection before it became an approved drug for such cases.

**Case presentation:**

A 56-year-old Kuwaiti woman with hepatitis C virus infection was treated with pegylated interferon α-2a and ribavirin, laboratory test results prior to therapy were within normal values. After 4 weeks of that treatment, she developed neutropenia and severe thrombocytopenia. Her hepatitis C virus treatment was stopped for many years until eltrombopag was used as an off-label drug with an episode of severe thrombocytopenia. Her platelets count returned to normal level when triple therapy for hepatitis C virus was used successfully.

**Conclusions:**

An off-label drug should be used only when it is the best available drug, based on evidence from on-going multicenter trials. It could be life saving for some patients in critical situations. However, clinical use of eltrombopag later confirmed that it is a safe and effective drug for immune thrombocytopenic purpura or thrombocytopenia associated with hepatitis C virus infection.

## Introduction

Hepatitis C virus (HCV) infection is known to result in thrombocytopenia, even in the absence of overt hepatic disease. This infection is considered a marker for severity of liver disease and may sometimes be the only manifestation of viral hepatitis. It is estimated that 160 million individuals worldwide have chronic HCV infection. Moreover, this infection has been associated with an increased risk of developing chronic immune thrombocytopenic purpura (ITP) [1]. HCV infection-induced thrombocytopenia has an underlying autoimmune mechanism similar to that of ITP. The virus binds to thrombocytes, resulting in the production of autoantibodies against thrombocyte membrane antigens. Over 90% of patients with chronic HCV infection develop high levels of immunoglobulin (Ig)G associated thrombocytes called platelet-associated IgG (PAIgG) [2]. The IgG antibodies react with specific glycoproteins on the thrombocyte membrane surface and label them for autoimmune destruction in the reticuloendothelial system [3]. High PAIgG levels are directly related with liver disease severity, suggesting that chronic HCV infection is associated with major changes in the immune system. Patients who underwent interferon-α and ribavirin therapy had significant adverse effects, including body aches, malaise in 60%, fever in 35%, anaphylaxis in 2%, thrombocytopenia in 15%, and granulocytopenia in 26% [4].

Off-label drug use (OLDU) refers to drug use for unlicensed indications, in terms of doses, preparation, patient population, or route of administration, beyond the specifications authorized for marketing [5]. In the USA, OLDU implies the prescription of a drug that is not specified in the labeling approved by the US Food and Drug Administration (FDA) [6].

## Case presentation

**Figure 1:**
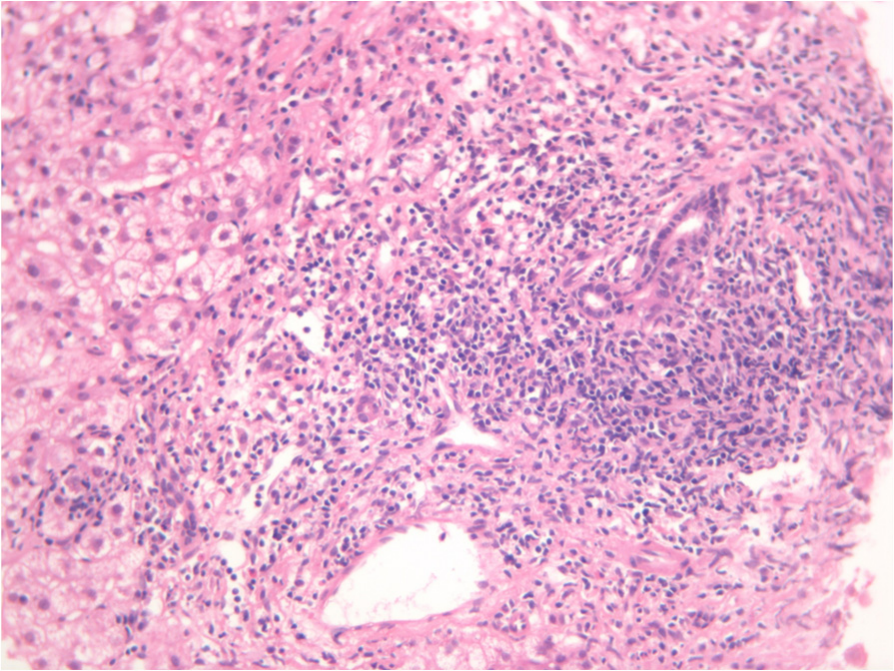
Inflamed portal tract area with interface hepatitis, shows inflammatory cells beyond the limiting plate.

In 2002, a 56-year-old Kuwaiti woman without a history of blood transfusion was diagnosed with chronic hepatitis C. She may have contracted the viral infection while administering insulin injections to a friend with chronic HCV. Virology studies revealed HCV genotype 1 infection. Polymerase chain reaction indicated an HCV-ribonucleic acid (RNA) viral load by TaqMan® of 785,000IU/mL (lower limit >15IU/mL). The histologic sections at that time showed the overall architecture was preserved. There was a portal and periportal fibrosis with occasional bridging. Histology also showed mild to moderate portal inflammation, composed of mostly small lymphocytes, with focal interface hepatitis. Few plasma cells and histiocytes were noted with mild to moderate lobular inflammation and scattered acidophil bodies and minimal macrovesicular steatosis. There was no significant cholestasis, Mallory hyaline, ballooning degeneration or intranuclear/cytoplasmic inclusions. Her central veins were unremarkable. Iron stain highlighted a few Kupffer cells with increase iron storage. Periodic acid–Schiff diastase stain was negative for intracytoplasmic globules (Figure 1).

In 2003, she was treated with pegylated interferon α-2a (Pegasys®, Hoffmann-La Roche Inc., NJ, USA) 180μg per week and ribavirin 1000mg daily. Laboratory test results prior to the therapy were as follows with the minimum and maximum normal values: white blood cell (WBC) count, 4 (4 to 10×10^9^/L); hemoglobin (Hb) level, 14.1g/L (12.0 to 15.0g/L); platelet count, 167×10^9^/L (150 to 410×10^9^/L); international normalized ratio, 1.1; total protein level, 72g/L (61 to 79g/L); albumin level, 42g/L (35 to 48g/L); alkaline phosphatase level, 66IU/L (42 to 98IU/L); alanine transaminase (ALT), 308IU/L (10 to 60IU/L); aspartate aminotransferase (AST) level, 174IU/L (10 to 42IU/L); and thyroid-stimulating hormone, 4.01mIU/L (0.43 to 4.1mIU/L). Her serum was negative for antinuclear antibodies, antimitochondrial antibodies and antismooth muscle antibodies. A quantitative assay indicated normal serum Ig levels. An abdominal ultrasound did not indicate any abnormality.

After 4 weeks of treatment with interferon, she developed neutropenia (WBC count, 2.9×10^9^/L). She was then administered 300μg of the granulocyte colony-stimulating factor Neupogen® (filgrastim; Amgen Inc., CA, USA). She also developed severe thrombocytopenia (platelet count, 5×10^9^/L) and underwent transfusion with 19 platelet units. A bone marrow biopsy revealed the presence of normal cellularity and normal differentiation of the three cell lineages, indicating neutropenia and thrombocytopenia due to peripheral destruction. Intravenous Ig 0.4g/kg daily was administered for 5 days. There was good response to the treatment, and her platelet count increased to 104×10^9^/L. Because of severe thrombocytopenia, she was considered unsuitable for further interferon therapy. Treatment with peginterferon had probably caused the thrombocytopenia, and thus, treatment was discontinued for approximately 7 years owing to the risk of thrombocytopenia recurrence. Between 2004 and 2010, her platelet count oscillated between 50 and 100×10^9^/L.

**Figure 2:**
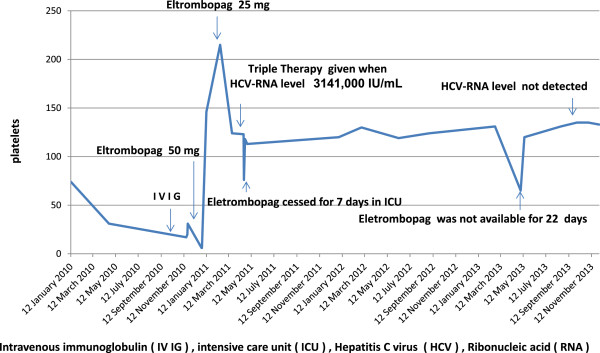
Platelets levels during the treatment course.

In late 2010, she again presented with severe and progressive thrombocytopenia. At that time, her platelet count dropped from 72 to 17×10^9^/L throughout the year. Intravenous Ig was administered without adequate response: platelets only increased to 31×10^9^/L and then dropped to 6×10^9^/L after 7 days of treatment. She was referred again to the Hematology Outpatient Consultation Department. Eltrombopag 50mg daily was administered off label because intravenous Ig became progressively less effective in this case. After 10 days of treatment, there was a very good response (Figure 2); her platelet count increased to 250×10^9^/L. The dose was then reduced to 25mg daily. After 3 months of normal platelet count by continuous administration of eltrombopag 25mg daily, the hepatologist considered the patient eligible for HCV infection treatment.

In 2011, an HCV-RNA viral load by TaqMan® of 3,141,000IU/mL was indicated; she received triple HCV infection therapy (interferon, ribavirin, and telaprevir). Her platelet count did not reduce while she was on eltrombopag as well as the triple therapy. However, after the triple therapy, she had serious complications and presented hemolytic anemia and urticaria. One month later, she developed severe pneumonia with oxygen desaturation and required mechanic ventilation in our intensive care unit for 7 days where eltrombopag was stopped. During this period, her platelet count decreased once again to 76×10^9^/L. However, once eltrombopag was resumed, her normal platelet count was re-established to normal values. She fully recovered from this complication and is currently leading a normal life with normal hematologic and normal serum chemistry parameters (Hb, WBC, platelet, ALT, AST, gamma-glutamyltransferase and so on). Even after 3.5 years, she continues to receive eltrombopag treatment. In 2013, her platelet count reduced to 67×10^9^/L owing to treatment cessation for 22 Days; her platelet count returned to 131×10^9^/L once she resumed taking eltrombopag 25mg daily (Figure 2). On May 2013 HCV-RNA viral load by TaqMan® was not detected.

## Discussion

Chronic HCV may be accompanied by variable levels of thrombocytopenia caused by different mechanisms: central and peripheral autoimmune mechanisms or drug-induced thrombocytopenia. An autoimmune mechanism was found in 85% of the cases. HCV infection can directly suppress megakaryocyte production. Interferon treatment also has a direct myelosuppressive effect that could lead to thrombocytopenia [7]. Patients are considered eligible for HCV infection treatment if their platelet count is above 90×10^9^/L [8]. In December 2012, eltrombopag was approved by the US FDA for treatment of HCV infection-related thrombocytopenia. It is the first drug approved for ITP treatment in patients who are refractory to other treatments (for example, corticosteroids, Igs) [9]. Eltrombopag is also the first orally bioavailable drug in its class and is a thrombopoietin receptor agonist that induces increased proliferation and differentiation of human bone marrow progenitor cells into megakaryocytes and increased platelet production in the circulation. The development of a targeted thrombopoietin receptor agonist has great implications for the therapy of patients with diseases associated with decreased platelets [10]. Eltrombopag is found in medical trials to provide clinical benefits in other conditions in which thrombocytopenia can occur, such as postchemotherapy and myelodysplastic syndrome [11]. Our patient did not refer or present any treatment related adverse effects on hematology, coagulation, or clinical chemistry parameters during her treatment with eltrombopag. Reportedly, the adverse effects of eltrombopag are mild and only seen in a few patients; these effects include headache, nasopharyngitis, upper respiratory tract infection, fatigue, arthralgia, diarrhea, nausea, back pain, and urinary tract infection [12]. OLDU based on evidence obtained from controlled research trials is a useful medical tool. This case is an example of life-saving treatment with OLDU, and this drug was approved after approximately 2 years for thrombocytopenia due to chronic HCV infection [13]. Currently, eltrombopag is in use in adult patients with HCV with chronic HCV infection for the treatment of thrombocytopenia when the degree of thrombocytopenia severity should be considered while maintaining optimal interferon-based therapy [14]. Platelet counts with eltrombopag treatment should be adjusted to maintain a platelets count of around 100×10^9^/L, to avoid the risk of portal vein thrombosis in a patient with liver disease [15].

## Conclusions

OLDU could be life saving for some patients in critical situations. Its use must be based on evidence from on-going multicenter trials. OLDU must be the best available drug to be used. However, not all off-label drugs become approved but the new thrombopoietin receptor agonist eltrombopag became an approved effective drug for thrombocytopenia associated with HCV.

## Consent

Written informed consent was obtained from the patient for publication of this case report and any accompanying images. A copy of the written consent is available for review by the Editor-in-Chief of this journal.

## Abbreviations

ALT: Alanine transaminase; AST: Aspartate aminotransferase; FDA: Food and Drug Administration; Hb: Hemoglobin; HCV: Hepatitis C virus; Ig: Immunoglobulin; ITP: Immune thrombocytopenic purpura; OLDU: Off-label drug use; PAIgG: Platelet-associated immunoglobulin G; RNA: Ribonucleic acid; WBC: White blood cell.

## Competing interests

The authors declare that they have no competing interests.

## Authors’ contributions

HA-J was the treating hematologist and wrote the manuscript. JA-K was one of treating hepatologists team, revised the manuscript and added references. AA is a histopathologist and reported the liver biopsy and revised the manuscript and added references. KA-B revised the manuscript and added references. All authors read and approved the final manuscript.
